# Health economic evaluation of forced orthodontic extrusion of extensively damaged teeth: up to 6-year results from a clinical study

**DOI:** 10.1007/s00784-023-05178-w

**Published:** 2023-07-27

**Authors:** Maria Bruhnke, Michael Naumann, Mats Wernfried Heinrich Böse, Florian Beuer, Falk Schwendicke

**Affiliations:** 1 Department of Prosthodontics, Geriatric Dentistry and Craniomandibular Disorders, Charité Universitätsmedizin Berlin, corporate member of Freie Universität Berlin, Humboldt–Universität zu Berlin and Berlin Institute of Health, Aßmannshauser Straße 4-6, 14197 Berlin, Germany; 2 Department of Oral Diagnostics, Digital Health and Health Services Research, Charité Universitätsmedizin Berlin, corporate member of Freie Universität Berlin, Humboldt–Universität zu Berlin and Berlin Institute of Health, Aßmannshauser Straße 4-6, 14197 Berlin, Germany

**Keywords:** Cost, Cost analysis, Cost-effectiveness, Forced eruption, Orthodontic extrusion, Tooth extrusion

## Abstract

**Objectives:**

Clinical data on retaining extensively damaged teeth using forced orthodontic extrusion followed by restorative rehabilitation are scarce, and economic evaluations are basically absent. A health economic evaluation of this method was performed based on a clinical study.

**Materials and methods:**

In a convenience sample of individuals recruited from routine care, extensively damaged teeth were orthodontically extruded prior to restoration. Patients were followed up for up to 6 years. The health outcome was tooth retention time. Direct medical, non-medical, and indirect initial and follow-up costs were estimated using the private payer’s perspective in German healthcare. Association of initial direct medical treatment costs and cofounding variables was analyzed using generalized linear models. Success and survival were secondary outcomes.

**Results:**

A total of 35 teeth in 30 patients were followed over a mean ± SD of 49 ± 19 months. Five patients (14%) dropped out during that period. Median initial costs were 1941€ (range: 1284–4392€), median costs for follow-up appointments were 215€ (range: 0–5812€), and median total costs were 2284€ (range: 1453 to 7109€). Endodontic re-treatment and placement of a post had a significant impact on total costs. Three teeth had to be extracted and in three patients orthodontic relapse was observed. The survival and success rates were 91% and 83%, respectively.

**Conclusions:**

Within the limitations of this clinical study, total treatment costs for orthodontic extrusion and subsequent restoration of extensively damaged teeth were considerable. Costs were by large generated initially; endodontic and post-endodontic therapies were main drivers. Costs for retreatments due to complications were limited, as only few complications arose.

**Clinical relevance:**

The restoration of extensively damaged teeth after forced orthodontic extrusion comes with considerable initial treatment costs, but low follow-up costs. Overall and over the observational period and within German healthcare, costs are below those for tooth replacement using implant-supported crowns.

**Trial registration:**

ClinicalTrials.gov Identifier: DRK S00026697).

## Introduction

The restoration of extensively damaged teeth (for example as a consequence of traumatic injuries or caries) remains controversial despite decades of research. The amount of residual tooth structure and the defect location have been shown to have a significant role in the long-term prognosis of both tooth and restoration [1]. Subgingival and subcrestal defects are particularly challenging as they usually violate the biologic width [2, 3] and as a circumferential ferrule design preparation is necessary for long-term success of post-endodontic restorations [4]. Hence, surgical crown lengthening [5] or orthodontic extrusion [6, 7] are two strategies employed prior to restorative procedures if extensively damaged teeth with such subgingival or subcrestal defects are to be retained.

Surgical crown lengthening is associated with esthetic and biologic disadvantages due to the lengthening of the clinical crown [8] and the osseous reduction of the alveolar bone level [9, 10], which is why forced orthodontic extrusion is increasingly discussed as a possible alternative [11, 12]. Orthodontic extrusion may be regarded a minimally invasive and maximally tissue preservative treatment procedure [13]. Alternatives to tooth retention involve extraction and (implant or otherwise supported) replacement of the tooth [14].

While there is a wealth of data towards the survival and prognosis of tooth-supported fixed dental prostheses [15] or implant-supported restorations [16], data on extensively damaged teeth which were orthodontically extruded and subsequently restored is extremely limited [11, 12]. In a recent clinical study, a survival rate of 94% was reported for extensively damaged teeth after forced orthodontic extrusion over a mean observation period of 3.3 years, and orthodontic relapse was demonstrated as the major complication of this treatment method [12].

Besides efficacy (longevity, survival), costs of a therapy are increasingly seen as a relevant aspect to consider during decision-making, as they are relevant for payers on all levels (patients, insurances, public funds) but also providers. For orthodontic extrusion and restoration of extensively damaged teeth, any such health economic data is largely absent, and hence any comparison with the discussed alternatives (tooth extraction and replacement) is impossible when it comes to economic aspects.

It was the aim of the present study to perform a health economic evaluation of orthodontically extruded and subsequently restored, extensively damaged teeth using routinely collected data of the discussed clinical study. The results of this study are of interest for patients, providers, and further decision-makers in dental healthcare.

## Materials and methods

### Study design

The clinical study was approved by the local ethics committee (approval number: EA2/301/20) and registered at the German Clinical Trials Register (DRKS registration number: DRK S00026697). Sample size calculation is described elsewhere [12]. We here report on a post-hoc health economic evaluation of the study cohort. Reporting of this study followed the Strengthening the Reporting of Observational Studies in Epidemiology (STROBE) [17] and the Consolidated Health Economic evaluation standard guidelines (CHEERS) [18]. All participants provided written informed consent.

### Sample and clinical procedures

Study participants were consecutively recruited at the prosthodontic department at Charité–Universitätsmedizin in Berlin. The following inclusion criteria were defined: patients with (1) permanent teeth with subgingival or subcrestal defect, with (2) two proximal contact points, (3) pocket probing depths of ≤ 2 mm, (4) defects violating the biologic width and lacking the option for ferrule design preparation, (5) a prospective crown-root ratio not exceeding 1, (6) tooth mobility ≤ I, and (7) expected to require a single crown restoration. Patients of 18 years and older were assessed for eligibility. Both endodontically treated as well as vital teeth were included. Radiographs were obtained before treatment to assess the apical condition of the tooth and to determine the need for endodontic treatment or retreatment. Teeth with a remaining residual dentin thickness at the orifice of the canal of more than 1 mm and a ferrule height of 2 mm after extrusion were included. Exclusion criteria were (1) teeth with hypercementosis or (2) vertical root fractures, (3) molars, (4) tooth mobility ≥ II, (5) probing depths of ≥ 3 mm, and (6) teeth with prospective crown-root ratios exceeding 1. Patients should have been willing to appear for follow-up appointment over the next 5 years.

**Fig. 1:**
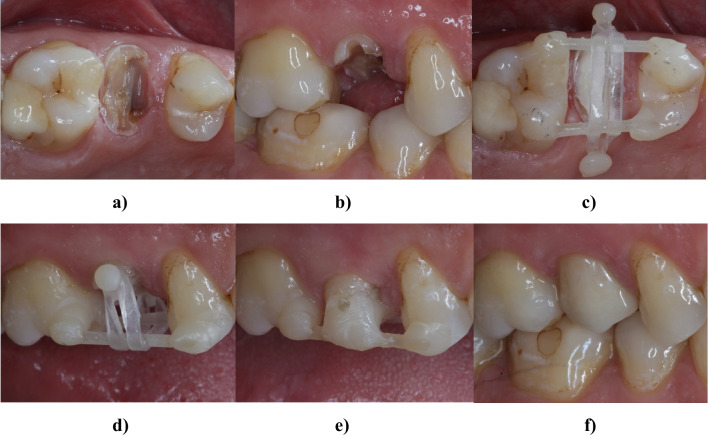
Initial clinical situation after removal of softened carious tissue from occlusally (**a**) and buccally (**b**). A composite-based bar was bonded to the root surface of the tooth to be extruded; a second bar served as anchorage on the adjacent teeth (**c**). Elastics were used to initiate movement in the occlusal direction (**d**). After successful extrusion, the tooth was splinted to adjacent teeth for retention (**e**). Final restoration after successful extrusion (**f**)

Figure 1 illustrates the clinical procedures for forced orthodontic extrusion; details are described elsewhere [19]. After removal of insufficient restorations and of all softened carious tissue, a fiber-reinforced composite-based bar (Extrusion pin, Komet Dental, Lemgo, Germany) was adhesively bonded to the root of the tooth to assist extrusion (RelyX Unicem 2 Automix, 3 M, Neuss, Germany). A second bar was luted onto the surface of the neighboring teeth using an etch-and-rinse adhesive (OptiBond FL, Kerr, Kloten, Schweiz) and a flowable composite (Tetric EvoFlow, Ivoclar, Schaan, Liechtenstein). Orthodontic elastics were used for occlusal movement of the root (> 0.5N). Supracrestal fiberectomy and scaling and root planning were performed [20]. Patients were instructed to change the elastics two times a day. After successful extrusion, teeth were adhesively bonded to the second bar and to the neighboring teeth with a flowable composite (Tetric EvoFlow) for a retention period of at least 8 weeks. After non-surgical re-treatment of existing root-canal fillings (if present), glass-fiber reinforced composite posts (X-Post, DentsplySirona, Bensheim, Germany) were placed with a self-adhesive resin cement (RelyX Unicem 2 Automix) in teeth with one or less residual coronal walls before final restoration (*n* = 24). For one tooth (central incisor), X-Post No3 (blue coding/1.67-mm diameter); for 15 teeth (4 lateral incisors, 3 central incisors, 2 canines, 6 premolars), X-Post No2 (red coding/1.47-mm diameter); and for 8 teeth (1 central incisor, 2 lateral incisors, 1 canine, 4 premolars), X-Post No1 (yellow coding/1.35-mm diameter) were used. Patients were contacted once a year during follow-up. Initial therapy procedures were performed under supervision of one operator (MB). Clinical and radiological re-examinations were performed by one operator (MB). Periodontal assessment was performed with the aid of a periodontal probe (HS-Parodontometer CP15; Henry Schein) under local anesthesia by the same operator. Radiographs were taken using paralleling technique. Analysis of radiographic images of extrusion and at recall is described elsewhere in detail [12].

### Data collection

For the present study, the following data were collected from electronic patient records: Employed restorative materials; initially performed restorative, endodontic, or periodontal therapies; possible restorative, endodontic, or periodontal re-treatments; and surgical or prosthetic retreatments including those leading to tooth loss and replacement. Furthermore, information on patients’ gender, age, and tooth type was collected.

### Health outcome

The health outcome was tooth retention in years, mainly as alternative outcomes (like oral-health-related quality of life) had not been measured, but also as the additional valuation of other health states (e.g., replaced tooth) is only limitedly possible at present [21]. Success and survival rates were secondary outcomes. Survival was defined as a tooth in situ at recall. Success was defined as a symptom-free tooth, with an intact restoration, no carious decay, normal pocket probing depths (< 3 mm), and no signs of orthodontic relapse, ankylosis, root resorption or periapical translucencies [12].

### Setting, perspective, and horizon

The study setting was German healthcare. A societal perspective was taken, reflecting on direct medical and non-medical costs as well as indirect costs (see below). In Germany, medical insurance is two-tiered, with most individuals (approx. 88%) being publicly insured (statutory insurance) and only a minority being privately insured. For members of the statutory insurance, nearly all dental procedures are fully covered, while only few need to be partially or fully paid out-of-pocket or by private (additional) insurances. Forced orthodontic extrusion and the associated procedures are usually not covered by the public insurance, which is why direct medical costs were estimated using the private payer’s perspective.

### Estimations of costs

Using the outlined itemized treatments provided, costs were estimated on the basis of fee items of the German private insurance catalog GOZ (Gebührenordnung für Zahnärzte) [22]. For GOZ, factoring of fee items is routine to determine individual costs reflecting complexity and time consumption of therapeutic procedures. In the present study, costs were calculated using the standard multiplication factor of 2.3; this is in line with previous research [23]. Calculation of costs with the aid of average national multiplication fees has shown no significant impact on cost-effectiveness [24]. Laboratory and material costs were similarly accounted for by using fee items. Costs estimations were subdivided in initial treatment costs and treatment costs for follow-up appointments for each tooth and patient individually, including costs for managing possible complications. Direct medical costs were calculated as the sum of all treatment and laboratory costs, including initial clinical and radiographical examinations, diagnostic models, forced orthodontic extrusion, post-and-core restorations, endodontic and periodontal procedures, final coronal restorations, as well as follow-up treatments. The number of appointments and patient’s addresses were derived from electronic records. Based on the patients address information in the electronic record, the number of kilometers was calculated per outward and return journey for one appointment. These travel costs were multiplied by a fixed fee per kilometers travel distance based on the German standard tax tariffs (0.3). These travel costs were defined as initial direct non-medical costs. Costs for time spent traveling and dental appointments were defined as indirect costs (opportunity costs). Calculation of time in minutes spend traveling was based on patient’s addresses. Time in minutes spent in dental appointments was derived from electronic calendars as well as time of photographic documentation. Due to a lack of primary data, indirect costs that were associated with patient’s waiting times or sick leave (e.g., after implantation) were not accounted for. Costs were multiplied by the average net hourly wage in Germany in each specific year of treatment. Costs were calculated both over the total period and per year of follow-up.

### Currency and discounting

Costs were calculated in 2016 Euros, assuming all patients have been initially treated in 2016 (which is when the cohort was established). Costs during follow-up were discounted at 3% per annum, accounting for time preference (money spent now being valued higher than money spent later) [25].

### Analytical methods

Costs were calculated using a spreadsheet (Excel, Microsoft, Redmond, USA). Initial treatment costs and costs for follow-up procedures were calculated individually. Quantitative parameters were described using medians, minima, and maxima and 25% and 75% percentiles. Association of total initial treatment costs and cofounding variables was analyzed using generalized linear models. The significance level was set up *p* < 0.05. Statistical analysis and data visualization were performed with the aid of SPSS (IBM, Armonk, USA) and Excel 2016.

## Results

A total of 30 patients (15 male and 15 female) with a mean ± SD age of 52.5 ± 18.9 years were included, and a total of 35 teeth were restored. The mean observation time was 49.1 ± 18.9 months. Five patients (14%) dropped out during follow-up: Four patients were not available for follow-up appointments due to no valid contact data, and one patient due to death.

**Fig. 2:**
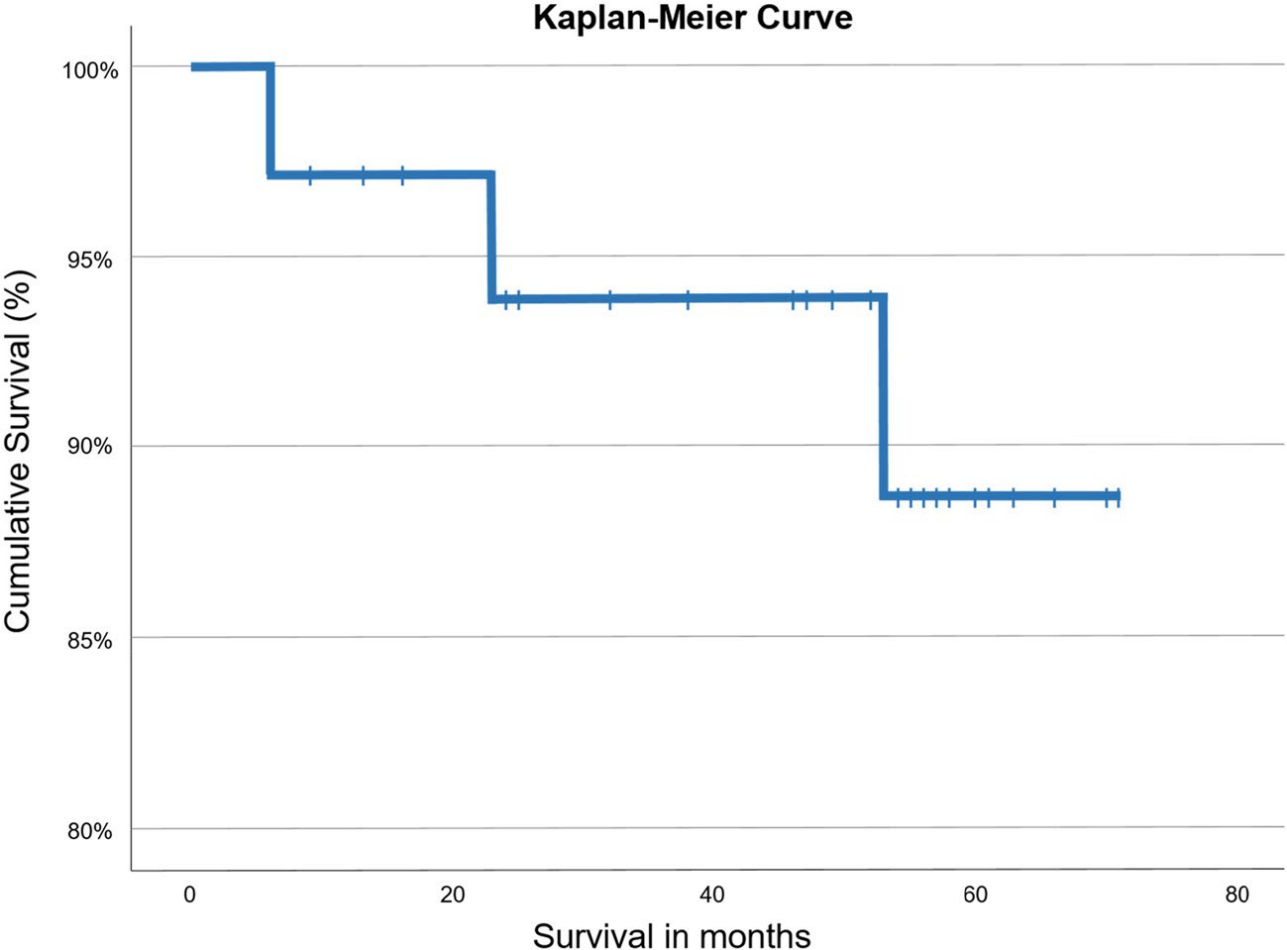
Cumulative survival for extensively damaged teeth after forced orthodontic extrusion

In six patients and six teeth, complications arose. (1) Three teeth in three patients had to be extracted due to fractures: One upper first premolar fractured in a patient with a history of bruxism. Furthermore, two lateral upper incisors fractured. For both teeth, a narrow diameter of the root became evident after extrusion due to the anatomical taper of the extruded tooth. (2) In three patients, some degree of orthodontic relapse was documented. However, as restorations remained intact, they were not renewed. This relapse was clinically acceptable to both patients and the operator. Thus, survival rate was 91% and success rate 83%. Figure 2 illustrates the cumulative survival rate. Table 1 provides on overview of all initial, follow-up, and total costs in Euro (€). Initial total costs were 1941.4€ ranging from 1283.5 to 4392.2€, follow-up total costs were 215.2€ ranging from 0 to 5812.1€, and overall total costs were 2283.6€ ranging from 1452.5 to 7108.6 €.

**Table 1:** Direct medical, direct non-medical, and indirect costs. Cost calculations are subdivided in subsections initial costs, costs for follow-ups, and total costs

Costs in Euro	Med.^1^	Min.^2^	Max.^3^	25% percentile	75% percentile
Total initial costs	1941.4	1283.5	4392.2	1747.1	2163.1
Initial direct medical costs	1604.6	1164	1887.4	1440.9	1741.6
Initial direct non-medical costs	66	8.1	772.2	36	92.4
Initial indirect costs	244	66.1	2179.1	160.8	369.4
Total costs after follow-up	215.2	0	5812.1	81	319.9
Direct medical costs at follow-up	97.7	0	5545.2	48.7	137
Direct non-medical costs at follow-up	23.6	0	244.1	7.8	36.9
Indirect costs at follow-up	85.1	0	578.3	26.9	125
Total costs	2283.6	1452.5	7108.6	1827.7	2565.9
Total direct medical costs	1765.9	1246.2	6710.5	1538.6	1922.4
Total direct non-medical costs	93.7	8.1	1016.3	39.1	141.1
Total indirect costs	346.7	128	2757.4	203.5	496.9

^1^Median, ^2^minimum, ^3^maximum

Mean tooth survival was 46 ± 20 months. In three patients, costs during follow-up were particularly high as teeth were extracted and replaced. For two patients, teeth were replaced with fixed dental prostheses, and total costs were 1595 and 1694.9€, respectively (initial direct medical costs: 86.4/28.8€; initial direct non-medical costs: 149.62/306.8€; initial indirect costs: 1359/1359.39€). One patient received an implant-supported single crown accounting to overall costs of 4827.5€ (initial direct medical costs: 4654.9€; initial direct non-medical costs: 9.6€; initial indirect costs: 163€).

Initial direct medical costs did not differ between gender and age. Tooth position did not have a significant impact on direct medical costs. However, endodontic treatment as well as re-treatment and placement of post had a significant impact. Table 2 provides the association of initial direct medical costs and covariates (gender, age, endodontic treatment, revision, post, tooth type, and jaw).

**Table 2:** Association between direct medical costs and covariates. Coefficients in Euro (Coeff.) and lower and upper confidence intervals (LCI/UCI) are provided; *p* values indicate significant differences

		Initial direct medical costs			
Parameter	Class	Coeff	LCI	UCI	Sig
Gender	Male	− 20.4	−115.7	74.8	0.663
	Female	Ref			
Age	< 50 years	85.5	−25.4	196.4	0.125
	> 50 years	Ref			
Jaw	Maxilla	−185.5	−395.7	24.7	0.081
	Mandible	Ref			
Tooth type	Anterior	26.9	−83.3	137	0.621
	Premolar	Ref			
Endodontic treatment	Yes	156.5	−55	368	0.141
	No	Ref			
Revision	Yes	225.8	116.3	335.4	** < 0.001**
	No	Ref			
Post	Yes	152.2	40	264.4	**0.010**
	No	Ref			

*SD*, standard deviation; bold values indicate significant differences

**Fig. 3:**
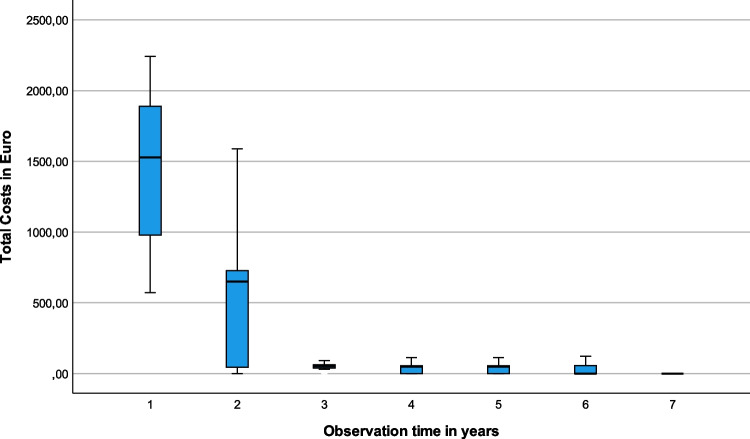
Box and whisker plots of total costs in Euro over the observation time per year after initial treatment. Assuming all patients have been initially treated in 2016, year 1 corresponds to 2016, etc.

Total costs were higher for initial treatment in comparison to follow-up. Figure 3 demonstrates the highest treatment costs in year one after initial treatment and decreasing costs afterwards.

## Discussion

This is the first study assessing treatment costs for retaining excessively damaged teeth using forced orthodontic extrusion and subsequent restoration. Overall, favorable survival rates and reasonable costs were observed for the majority of teeth. Two lateral incisors and one upper premolar had to be extracted due to fractures and were defined as failures. Notably, these teeth generated high costs for retreatment, mainly as replacement was expensive. Overall, costs were mainly generated initially. The findings of the present study need more in-depth discussion.

First, according to the analysis, retaining excessively damaged teeth using forced orthodontic extrusion and subsequent restoration is likely to be less costly initially than tooth removal and subsequent implant placement in the chosen healthcare setting and perspective, where implant-supported crowns come with initial direct medical costs of around 4700€. Costs are comparable to a calculation using the national fee averages from the 2009 American Dental Association survey of dental fees [26]. The findings corroborate a recent EAO consensus conference narrative review which concluded that, for example, retaining central incisors with irreversible pulpitis and coronal lesions using root canal treatment was more cost-effective than implant-supported single crowns [27]. A similarly high cost-effectiveness of tooth retention has been demonstrated in an health economic evaluation using a US perspective [26] and a range of other studies [28, 29]. Notably, none of these evaluations focused on deeply destroyed teeth; the present study now adds a piece to this jigsaw. Also note, however, that both clinical success, treatment needs, and the resulting cost-effectiveness will be heavily determined by a wide range of clinical factors, like restorability, quality of bone and overall dental conditions, the individual esthetic demands, and general health conditions [30]. Moreover, cost estimations highly depend on the method of extrusion as it may be achieved by various ways, e.g., banding full arches and applying occlusal forces with the aid of elastics [31], applying magnetic extrusion procedures [32], as well as the use of removable orthodontic appliances [33] or existing removable partial dentures [34]. Moreover, surgical extrusion may be regarded as an alternative technique [35, 36]. This method might possibly result in rather low opportunity costs at initiation of treatment due to a minimized number of visits.

Second, and along these lines, costs were associated with a range of aspects, most importantly the need for endodontic retreatment or post placement. Both, endodontic retreatment and post placement significantly influenced cost estimations. These findings are comprehensive as both treatment procedures are expenses that play a role in cost analysis in addition to the actual procedures of forced orthodontic extrusion. When informing patients towards treatment decisions, these aspects should be borne in mind. Patient’s age, gender, tooth type, and type of jaw did not influence treatment costs significantly. As gender and patient’s age did not affect tooth survival in long-term clinical trials investigating different post systems [37], these findings are not surprising. Notably, the finding that anterior teeth are at higher risk of complications due to high shear forces compared to the posterior zone was not confirmed by results of the present study [1]. However, data are in line with another clinical study on teeth requiring forced orthodontic extrusion [11] and describing orthodontic intrusion as a major complication of this technique.

Third, if complications arose, retreatment costs were considerable, mainly as tooth replacement was cost-intense. It is conceivable that failure risk increases over time, which is why even longer follow-up periods than the one in this study are needed to establish sound estimates of failure risk. Moreover, it may be that under a worst-case scenario, immediate tooth removal and replacement could be more cost-effective than the costly tooth retention via orthodontic extrusion. Comparative analyses are required to assess this relative cost-effectiveness ranking.

This study has a number of strengths and limitations. First, it is the first study focusing on the economic evaluation of retaining teeth using orthodontic extrusion. Moreover, cost estimates reflected the true costs to payers and society in Germany. Second, and as a limitation, these costs may not apply to other settings and health care systems given the specific way they were derived. Third, our sample size was small, which may impact on the robustness of our cost estimates, but also the power to detect significant associations of various cofounding variables and costs. Fourth, treatment protocols were highly standardized, and all teeth were managed by the same dentists; clinical success but also costs may differ if other operators are considered (as they may perform differently, but also employ different restorative and/or surgical approaches). Moreover, the applied extrusion set described in the present study is currently no longer available for purchase. Therefore, a modified workflow with glass-fiber reinforced composite posts supplied by another manufacturer will be necessary in future research. Overall, and given the nature of this study, our findings should be interpreted with caution and require further confirmation.

## Conclusion

Within the limitations of this clinical study, total treatment costs for forced orthodontic extrusion and subsequent restoration of extensively damaged teeth were considerable. Costs were by large generated initially; endodontic and postendodontic therapies were main drivers. Costs for retreatments due to complications were limited as only few complications arose. Comparative cost-effectiveness evaluations are needed to establish economic rankings of different management strategies (including tooth removal and replacement) for extensively damaged teeth.
